# Dynamic Set Planning for Coordinated Multi-Point in B4G/5G Networks

**DOI:** 10.3390/s21051752

**Published:** 2021-03-03

**Authors:** Jia-Ming Liang, Ching-Kuo Hsu, Jen-Jee Chen, Po-Han Lin, Po-Min Hsu, Tzung-Shi Chen

**Affiliations:** 1 Department of Electrical Engineering, National University of Tainan, Tainan 70005, Taiwan; jmliang@mail.nutn.edu.tw (J.-M.L.); cliffabc2004@gmail.com (P.-H.L.); 2 Department of Information Management, Chung Cheng University, Chiayi 621301, Taiwan; mikehsu781122.cs03g@nctu.edu.tw; 3 College of Artificial Intelligence, National Chiao Tung University, Hsinchu 30010, Taiwan; 4 College of Artificial Intelligence, National Yang Ming Chiao Tung University, Hsinchu 30010, Taiwan; 5 Department of Computer Science, National Yang Ming Chiao Tung University, Hsinchu 30010, Taiwan; wenger0815@gmail.com; 6 Department of Computer Science and Information Engineering, National University of Tainan, Tainan 70005, Taiwan; chents@mail.nutn.edu.tw

**Keywords:** coordinated multi-point (CoMP), cooperating set, dynamic cell selection (DCS), energy efficiency, beyond fourth-generation/fifth-generation (B4G/5G), resource allocation, soft frequency reuse (SFR)

## Abstract

*Coordinated Multi-Point (CoMP)* is an important technique in B4G/5G networks. With CoMP, multiple base stations can be clustered to compose a *cooperating set* to improve system throughput, especially for the users in cell edges. Existed studies have discussed how to mitigate overloading scenarios and enhance system throughput with CoMP statically. However, static cooperation fixes the set size and neglects the fast-changing of B4G/5G networks. Thus, this paper provides a full study of off-peak hours and overloading scenarios. During off-peak hours, we propose to reduce BSs’ transmission power and use the free radio resource to save energy while guaranteeing users’ QoS. In addition, if large-scale activities happen with crowds gathering or in peak hours, we dynamically compose the cooperating set based on instant traffic requests to adjust base stations’ BSs’ transmission power; thus, the system will efficiently offload the traffic to the member cells which have available radio resources in the cooperating set. Experimental results show that the proposed schemes enhance system throughput, radio resource utilization, and energy efficiency, compared to other existing schemes.

## 1. Introduction

Cellular networks have evolved from providers of voice service ubiquitous coverage to available access ports anytime-and-anywhere for large bandwidth data services in the last decade. The number of mobile devices per holder is increasing in the incoming three years. Billions of low-data-rate machine-to-machine (M2M) devices with cellular connectivity are predicted to be deployed and operated in the near future. Subsequently, we are facing the *1000x* data challenge or capacity crunch. Moreover, with increasing mobile subscribers and increasing information technology contribution to the whole energy consumption, it is necessary to reduce the radio access energy requirements without significantly compromising users’ experience of the *quality of service (QoS)* [1].

To support many devices to access wireless networks simultaneously, 3GPP release 15 [2] is deployed to support higher data rates for users. Additionally, many new techniques, such as *Coordinated Multi-Point (CoMP)* [3,4,5], relaying networks, and cells collocated in the B4G/5G networks are proposed to increase system throughput and satisfy devices’ requirements. This paper investigates the issue of CoMP and energy efficiency in B4G/5G networks, where CoMP is used to maximize throughput of a cell in cell center and cell edge, and for energy-efficiency, explores how to effectively allocate base stations’ BSs’ transmission power to the serving areas such that the consumed energy per bit can be minimized.

In this paper, we propose to leverage adaptive power allocation and the dynamic CoMP mechanism for balancing load and saving energy in B4G/5G networks. During the off-peak time, our proposed method will enter power saving mode to lower the transmission power of BSs and guarantee users’ QoS. If crowd-gathering activities happen, we exploit a dynamic cooperating set concept with adapted transmission power for tackling the overloading problem. When an overloaded BS appears in the system, the overloaded base station as the center of a cooperating set invites adjacent cells with free resources to join and form the cooperating set, so as to mitigate the load of the overloaded BS and meet the QoS requirements of the users. Adaptive transmission power allocation and dynamic inter-/inner-cell selection will also be executed in an iterative manner to enhance the energy efficiency and frequency resource utilization, and offload more users in the overloaded cells.

The contributions of this paper are three-fold. First, this is the complete work addressing the issue of dynamic set planning in B4G/5G networks. Since B4G/5G applies a small cell scenario, the dynamic set planning issue becomes more important [6]. Second, we propose a scheme considering both off-peak hours and overloading scenarios, which is realized in three sub-algorithms: (1) Dynamic cooperating set planning (DCSP) sub-algorithm, where the main idea is to invite one-hop and two-hop neighbors to form an efficient cooperating set. (2) Power-saving resource allocation (PSRA) sub-algorithm, which adaptively adjusts transmission power to enhance throughput. (3) Intra-cell dynamic service area selection (ICDSS) sub-algorithm: to further enhance throughput and capacity offloading, where the idea is to allocate free resources for the cell inner region first and make the cell outer area get more resources to help with offloading. Third, the performance of the proposed scheme is verified to significantly enhance system throughput, radio resource utilization, and serve more users.

The rest of this paper is organized as follows. Related work is discussed in Section 2. System model and problem definition are given in Section 3. Section 4 presents our proposed scheme. Simulation results are given in Section 5. Conclusions are drawn in Section 6.

## 2. Related Work

In the literature, an important mechanism in the future network is the *Coordinated Multi-Point (CoMP)*, which has two categories. One is *Coordinated Scheduling/Coordinated Beamforming (CS/CB)* [7,8,9], where the data destined to a user is saved only in its serving cell and the scheduling of resources and beamforming are co-determined by the cooperating set. The other is *Joint Processing (JP)*, where the data destined to a user is saved in each cell of the cooperating set and the resource scheduling is co-determined by the cooperating set. JP can be further classified into two types with consideration of the transmission mode: *Joint Transmission (JT)* [10,11] and *Dynamic Cell Selection (DCS)* [12,13]. The first one transmits data to users by coordinating several base stations (BSs) for transmitting simultaneously, thus enhancing users’ reception signal quality. The second one lets the cooperating set dynamically choose users, while the BS does not need to be the serving cell of the users. Recently, several researchers have discussed the DCS and cooperating set. The work of [14] studies the control plane protocols for cooperative communications and proposes a novel coordination architecture to enhance the performance of multi-cell cooperative cellular networks. The study of [15] proposes a Poisson-Delaunay-triangulation-based method to allow the cooperating BS set of the *user equipment (UE)* to be fixed and off-line as determined by the BSs’ location information. The research of [16] proposes a deep-learning-based scheme to enhance the throughput of the DL CoMP in heterogeneous 5G NR networks. Addressed in [17,18,19] is the issue of latency between cooperating cells. The work of [17] proposes a throughput-aware controller placement method in optical metro networks. The research of [18,19] proposes coordinated scheduling methods for Time Division Multiplexing Passive Optical Networks. The work of [17,18,19,20,21] considers traffic scheduling under a DCS C-RAN. The work of [17] designs a dynamic bargaining-based approach to enhance throughput. The study of [18] proposes a scheduling method with embedded mode selection criteria for LTE-WLAN aggregation to enhance the aggregation ratio. The work of [19] proposes a Q-learning-based cell selection algorithm in sparse mobile crowdsensing to reduce data collection costs. The study of [20] designs an evolutionary-game-based scheme to improve secondary transmitters’ utility. The research of [21] proposes a matching method to reach a near-optimal result. However, all the above mentioned literature [17,18,19,20,21] does not address the energy efficiency issue. Our work considers how to form a cooperating set dynamically and how to adapt transmit power to maximize energy efficiency while guaranteeing users’ QoS at the same time.

## 3. System Model and Problem Definition

### 3.1. System Model

**Figure 1:**
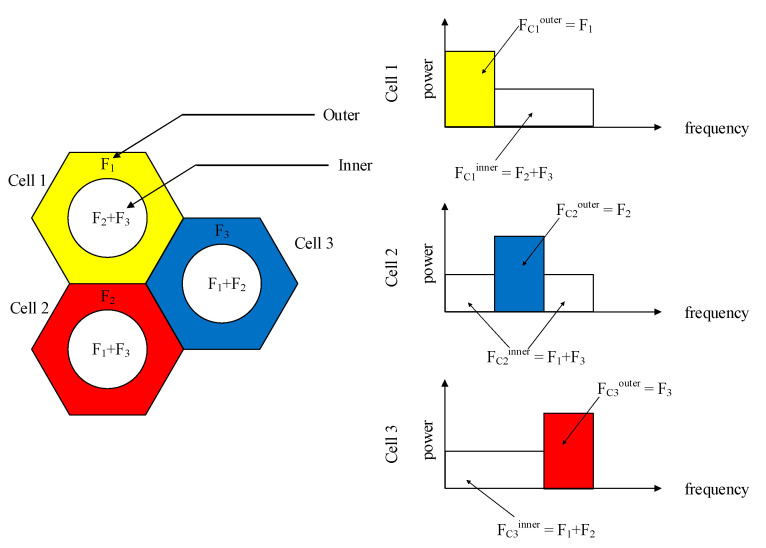
SFR model.

3GPP applies an *OFDMA (Orthogonal Frequency Division Multiple Access)* technique for wireless transmission. In the OFDMA system, simultaneous data transmission activities occur if the UE uses the same frequency bands. Next, this work exploits the *soft frequency reuse (SFR)* model [22,23,24,25] to reduce *ICI (Inter-carrier Interference)* to increase frequency efficiency. Figure 1 shows the SFR model, where each of the 3 cells has a frequency reuse unit for both center and edge areas. The frequency band is split into 3 subbands, F_1_, F_2,_ and F_3_, where each cell edge in a frequency reuse unit uses different frequency subbands, F_1_, F_2,_ or F_3_, while each cell center exploits the frequency subbands different from its cell edge, for example, the edge area of cell 2 uses subband F_2_ but the center area uses F_1_ and F_3_. To efficiently reduce ICI, subbands of centers are allocated low power while the subband of edges is allocated high power. Therefore, the system can mitigate ICI.

The area of the cell edge is covered by multiple cells. If the resource of one cell is not sufficient, we perform offloading by transmitting edge UEs to the neighboring cells with DCS. Previous works apply a fixed cooperating set [15]. Though these methods are easy to work with, they are not flexible and also cannot adjust the set to fit a real traffic scenario. Thus, we design the dynamic cooperating set concept. Based on the real traffic scenario, the proposed scheme dynamically invites proper neighboring cells to compose a cooperating set and then more users will be serviced with better radio resource utilization.

**Figure 2:**
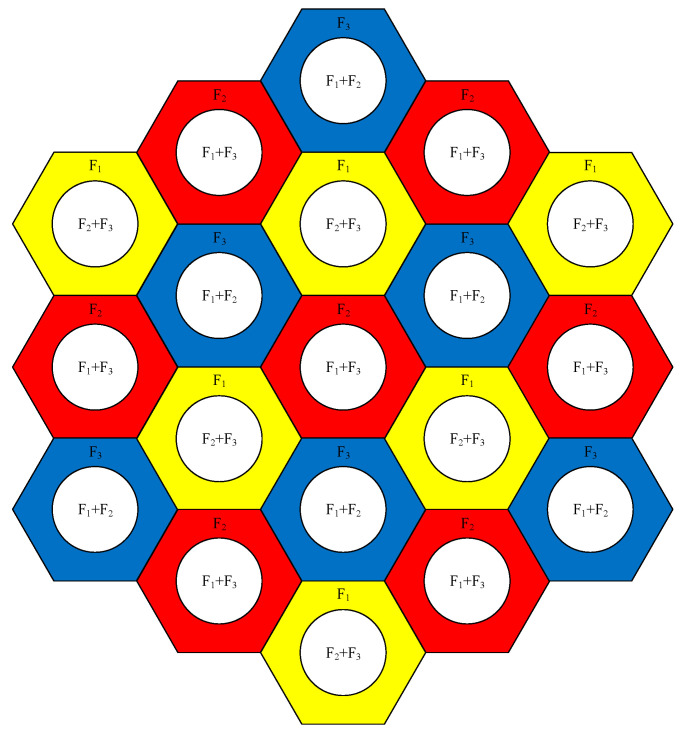
Deployment of base stations and channel assignment.

We try to tackle the following problem in this paper. Each cell in the system uses an omnidirectional antenna, and the cell edges share 1/3 area of the total area. Deployment of base stations and channel assignment is shown in Figure 2. Initially, each cell is responsible only for the users within its coverage area (for the users covered by several cells, they choose the closest one as the serving cell). If a cell or several adjacent cells overload, these cells will become the cluster head (or head of the cooperating set) and start to invite adjacent cells with available resources to join the cluster. The cooperating set size is not fixed, but the number of hops for cooperating cells is limited to ensure the delay for exchanging information among cooperating cells. Based on the real traffic situation, the cooperating set is dynamically regulated. Here, we are to find suitable cells to join the cooperating set. For a larger size cooperating set, the overall resources can be more effectively used, but the computing overhead is high. Our observation also shows that in some cases, we can add more cells into the cluster to alleviate the overloading problem and satisfy more users but the overloading problem cannot always be solved.

We apply the Signal-to-Interference-plus-Noise Ratio (SINR) model to evaluate channel quality. According to the SINR received, the system chooses a proper *modulation coding scheme (MCS)* for each UE. Table 1 illustrates MCS and the required received SINR.

### 3.2. Problem Definition

In this paper, we consider a network with *M* cells and *N* UEs. Each *UE_i_*, *i* = 1..*N*, has an average data rate of *δ_i_* bits/s. Most of the radio resource is free in off-peak hours. We focus on reducing the output power of BSs while guaranteeing the QoS of users. Therefore, the energy consumption of BSs will be saved and the radio resource utilization will also be enhanced. On the other hand, if during peak hours or when a crowd-gathering event happens, we dynamically adjust the cooperating set by inviting the neighboring BSs based on the real traffic scenario and the neighbor BSs’ status. With the available resources of neighbor BSs, we can balance loads with DCS and power adaptation so that more users will be serviced. We also significantly enhance system throughput and resource utilization. The notations used in this paper are summarized in Table 2.

## 4. The Proposed Scheme

In this work, we consider both off-peak and peak hours. During off-peak hours, the proposed scheme exploits the *power-saving resource allocation sub-algorithm* with scheduling resources and power with an energy-efficient view. If a crowd-gathering event happens, we exploit offloading by a dynamic cooperating set mechanism, DCS, and power adaptation. The offloading scheme has three sub-algorithms, *dynamic cooperating set planning sub-algorithm*, *transmit power allocation sub-algorithm*, and *intra-cell dynamic service area selection sub-algorithm*.

### 4.1. Power-Saving Resource Allocation (PSRA) Sub-Algorithm

Undoubtedly, energy efficiency is an extremely popular issue nowadays. This subsection focuses on resource allocation with power-saving during off-peak hours. During off-peak hours, most radio resources are unutilized. We consider reducing BSs’ output power on the premise that the QoS of users can be guaranteed. Thus, we can both reduce BSs’ energy consumption and enhance radio resource utilization.

In the following, we show the energy-saving resource allocation scheme step-by-step during off-peak hours. At the start, each BS is an independent and single-cell cooperating set. First, we calculate each user *j*’s required TTIs based on its request *r_j_* and calculate the channel condition of each user and interference with existing mechanisms. The inner transmission power and outer transmission power of BS *i* are *TP_i_^inn^* and *TP_i_^out^* (watt/TTI), respectively. Each BS *i* allocates radio resource to service user *j* based on its request *r_j_* and located area; that is, if *j* is in the inner region, *j* needs resource of *TTI^inn^_(i,j)_*; on the other hand, if *j* is in the outer region, *j* needs resource of *TTI^out^_(i,j)_*. To evaluate *TTI^inn^_(i_,_j)_* or *TTI^out^_(i,j)_*, we calculate SINR of *j*, and we exploit the available modulation and coding scheme *MCS^inn^_(i,j)_* or *MCS^out^_(i,j)_* by SINR, the *TTI^inn^_(i,j)_* and *TTI^out^_(i,j)_* are derived as follows:

(1) $$TTI_{\left(i,j\right)}^{inn}=\left\lceil \frac{r_{j}}{Eff\left(MCS_{\left(i,j\right)}^{inn}\right)}\right\rceil ,$$

(2) $$TTI_{\left(i,j\right)}^{out}=\left\lceil \frac{r_{j}}{Eff\left(MCS_{\left(i,j\right)}^{out}\right)}\right\rceil .$$

The main idea of TTI is the time length required to carry efficient data for service. Note that *Eff(x)* with MCS *x* is the number of data bits that a TTI can carry. Thus, the total requirements of BS *j* for inner and outer regions are derived as follows:

(3) $$TTI_{i}^{inn}=\sum_{j=1}^{N_{i}}TTI_{\left(i,j\right)}^{inn},$$

(4) $$TTI_{i}^{out}=\sum_{j=1}^{N_{i}}TTI_{\left(i,j\right)}^{out},$$

which is the sum of the requirements of all users, where *N_i_* is the number of users served by BS *i*. We determine lower and upper thresholds for both inner and outer regions. The lower and upper thresholds of the inner region are *Thr^inn^_lw_* and *Thr^inn^_up_*, respectively, while those of the outer region are *Thr^out^_lw_* and *Thr^out^_up_*, respectively. Based on the relation between *TTI_i_^out^* and *TTI_i_^inn^* to these thresholds, we adjust BS *i*’s transmission power for energy-saving and enhance resource utilization. If *TTI_i_^inn^* < *Thr^inn^_lw_* or *TTI_i_^out^* < *Thr^out^_lw_*, the base station will reduce *TP_i_^inn^* or *TP_i_^out^* to reduce energy consumption and enhance resource utilization until *TP_i_^inn^* ≥ *Thr^inn^_lw_* or *TP_i_^out^* ≥ *Thr^out^_lw_*, respectively.

If *TTI_i_^inn^* > *Thr^inn^_up_* or *TTI_i_^out^* < *Thr^out^_up_*, in order to avoid the base station suffering a sudden traffic burst and be overloaded at the next moment, the base station will increase *TP_i_^inn^* or *TP_i_^out^* until *TTI_i_^inn^* ≤ *Thr^inn^_up_* or *TTI_i_^out^* ≤ *Thr^out^_up_*, respectively. Note that the total transmit power cannot exceed the maximum transmission power *TP_MAX_* constraint, i.e,

|*S_i_^inn^*| ∗ *TP_i_^inn^* + |*S_i_^out^*| ∗ *TP_i_^out^* ≤ *TP_MAX_*, (5)

which means that the transmission power of all used TTIs cannot exceed the maximum transmission power *TP_MAX_* constraint, where *S_i_^inn^* and *S_i_^out^* are the sets of used TTIs in both inner and outer regions. After the BS updating *TP_i_^inn^* and *TP_i_^out^*, users will get new SINR and be re-allocated TTIs. So the sub-algorithm must be re-executed.

If the base station *i* has reached *TP_MAX_*, but *TTI_i_^inn^* and *TTI_i_^out^* are still greater than the threshold *Thr^inn^_up_* and *Thr^out^_up_*, we will not update them in order to ensure *TP_MAX_* constraint. When the radio resource of the base station is not sufficient to meet users’ requirements, the dynamic cooperating set planning scheme will be executed, which will be illustrated in the next subsection.

### 4.2. Dynamic Cooperating Set Planning (DCSP) Sub-Algorithm

**Figure 3:**
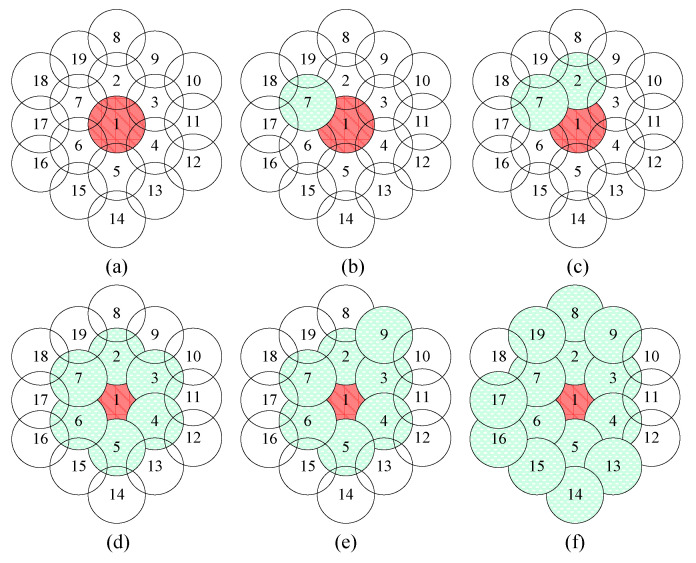
An example of dynamic cooperating set planning.

This subsection illustrates the way of dynamically composing the cooperating set for offloading traffic. At the start, each BS operates independently. Consider a case that some BSs in the system have a crowds-gathering activity, and traffic request is overloaded, which causes many users to fail access to the network. To handle this problem, we propose a method that dynamically composes the cooperating set by inviting the BSs adjacent to the overloaded areas according to real traffic conditions and neighbor BSs’ status. Thus, the cooperating set efficiently disperses the overloading traffic demand to the BSs with available resourced with DCS, and increases spectrum efficiency, and satisfies more UEs. The algorithm has two parts. First, consider the adjacent BSs to the overloaded BS (*C_h_*), called one-hop neighbors of *C_h_*, denoted by *ψ^1^_Ch_* = {*N^1^_i_*, *i* = 1..6}, *N^1^_i_* is the *i-th* direct neighboring cell of *C_h_*. To reduce loading of *C_h_*, the users that are originally served by *C_h_* and are covered by any neighboring BS in *ψ^1^_Ch_* are dynamically scheduled to exploit available resource of cells in *ψ^1^_Ch_* by DCS; that is, transferring part of the edge users from *C_h_* to neighboring BSs, *N^1^_i_*, *i* = 1, …, 6. If overloading still exists after all *N^1^_i_* ∈*ψ^1^_Ch_*, *i* = 1, …, 6, join the cooperating set, the method enters the second stage. Here, we consider BSs that are neighboring to the cooperating set where they are not next to BSs of *C_h_* but two-hop neighbors of *C_h_*. Next, we continue choosing proper BSs to join the cooperating set. These BSs can *relay* their resource to *N^1^_i_*, *i* = 1, …, 6, by serving *N^1^_i_’s* edge users, hence increasing the available resource of *N^1^_i_* that can be used to offload more users in *C_h_*. An example for planning a dynamic cooperating set is shown in Figure 3. Specifically, Figure 3a shows an overloading scenario that occurs in cell 1, which triggers the procedure. First, cell 1 asks the one-hop neighbors to join the cooperating set, as shown in Figure 3b–d, for supporting offloading. If the one-hop neighbors are not sufficient to tackle the overload scenario, the cooperating set will invite two-hop neighbors, as shown in Figure 3e,f, to join the cooperating set. Then, it will reach load balance and enhance spectrum efficiency and throughput.

#### 4.2.1. Including One-hop Neighbors (ψ^1^_ch_)

This first part selects a suitable directly adjacent BS for the cooperating set (in the rest of the article, we use a cluster instead of a cooperating set, denoted by *G*). We select BSs to join cluster *G* by considering the following three parameters, *α_m,n_*, *β_u,v_* and *F(C^1^_i_, G)*, where *α_m,n_* is the set of users which is served by BS *m* and is also covered by BS *n*, *β_u,v_* is the amount of free resource in the cell edge of BS *v* which can be provided to BS *u* and *F(C^1^_i_, G)* is to calculate the number of adjacent edges between BS *C^1^_i_* and *G*. With *α_m,n_*, *β_u,v_*, and *F*(*C^1^_i_, G*), we define and calculate the weight W*_i_* of base station *C^1^_i_* as follows, which is the weighted average of *α_m,n_*, *β_u,v_* and *F(C^1^_i_, G)*:

*W_i_* = *X* ∗ *α_ch,i_* + *Y* ∗ *β_ch,i_* + (1 − *X* − *Y*) ∗ *F(C^1^_i_, G)*, (6)

A larger α*_ch,i_* indicates that *C_h_* has more potential to transfer its edge demand to BS *C^1^_i_*, while a larger *β_ch,i_* means that BS *C^1^_i_* has more TTI in the cell edge. *F(C^1^_i_, G)* evaluates the overlapping area between BS *C^1^_i_* and cluster *G*. The greater the overlapping area, the more potential resource can be borrowed to cluster *G*. Carefully observing the relationship among *α_ch,i_*, *β_ch,i_* and *π* (*π* is the total amount of overloaded traffic demand of *C_h_*), we can find the following features: A1: When *π > Z(α_ch,i_)* (Z*(α_ch,i_)* represents the total required amount of radio resource in C*_h_* for the set of users *α_ch,i_*), which means that even if the base station *C^1^_i_* has sufficient resource, the overloading problem is still unable to be resolved; A2: When *π* ≤ *Z(α_ch,i_)*, if there exists a subset *α^h^_ch,i_* ∈ *α_ch,__i_* such that *π* ≤ *Z(α^h^_ch,i_)* and *β_ch,i_* ≥ *Z^b^_ch,i_*(*α^h^_ch,i_*) are true, where *Z^b^_ch,i_*(*α^h^_ch,i_*) represents the total amount of required TTIs in *C^1^_i_* for the set of users *α^h^_ch,i_*, then *C^1^_i_* can provide enough resource to solve the overloading problem; B1: When *β_ch,i_* < *Z^b^_ch,i_*(*α_ch,i_*), which means that it is possible for *C^1^_i_* to relay resource of *C_h_*’s two-hop neighbors to increase *β_ch,i_* to help offloading; B2: If *β_ch,i_* ≥ *Z^b^_ch,i_*(*α_ch,i_*), which means that it is impossible for *C^1^_i_* to use up its free resource or relay more resource from *C_h_*’s two-hop neighbors to offload more users in *C_h_*. Below, we illustrate how to select base stations in *ψ^1^_ch_* to join cluster G step by step.

**Step1**. If some cell *C_h_* overloads, our scheme starts executing.

**Step2**. *G* = {*C_h_*}, *A* = Ø, *S* = Ø and calculate *π*. Consider adding directly adjacent cells of *C_h_* into *G* first, i.e., set *S* = {*C^1^_i_*, *i* = 1, …, 6}. Calculate *α_ch,i_*, *β_ch,i_*, *Z(α_ch,i_)*, *Z^b^_ch,i_(α_ch,i_)*, and *W_i_* for each *C^1^_i_* ∈ *S*.

**Step3**. Select the cell C^1^_i__*_ in *S* with the greatest *W_i_* to join *G.* Update *G* = *G*+ {C^1^_i__*_} and *S* = *S*-{C^1^_i__*_}.

**Step4**. If *π*, α_ch,i__*_, Z(α_ch,i__*_) and β_ch,i__*_ meet the circumstance A2, terminate the algorithm and stop organizing the cooperating set. *G* is the final cooperating set; otherwise, go to step 5.

**Step5**. If the relationship of *π*, α_ch,i__*_, Z(α_ch,i__*_), Z^b^_ch,i*_(α_ch,i__*_) and β_ch,i__*_ confirm condition B1, which means that C^1^_i__*_ can only provide limited free resource, but it is still possible for C^1^_i__*_ to relay free resource from the outer BSs (*C_h_*’s two-hop neighbors) to increase β_ch,i__*_ to solve the overload problem. Update *A* = *A*+{C^1^_i__*_}, If the relationship of *π*, α_ch,i__*_, Z(α_ch,i__*_), Z^b^_ch,i*_(α_ch,i__*_), and β_ch,i__*_ confirm condition B2, which means that C^1^_i__*_ cannot help to solve the overloading problem even if it relays free resource from the outer BSs, so do not add C^1^_i__*_ into set A.

**Step6**. Find out a subset of α^h’^_ch,i__*_ ∈ α_ch,i__*_, where α^h’^_ch,i__*_ = *arg* max{ Z(α^h^_ch,i__*_)| Z^b^_ch,i*_(α^h^_ch,i__*_) ≤ β_ch,i__*_, α^h^_ch,i__*_ ∈ α_ch,i__*_}, transfer the set of users *α^h’^_ch,i_* to the base station C^1^_i__*_ and update α_ch,i__*_, β_ch,i__*_, and *π*.

**Step7**. For each *C^1^_i_* ∈ *S*, update *α_ch,i_*, *β_ch,i_*, *Z(α_ch,i_)*, *Z^b^_ch,i_(α_ch,i_)*, and *W_i_*. Update *π*, too.

**Step8**. If *S* is empty, go to step 9; otherwise, go back to step 3.

**Step9**. If *π* ≠ 0 and *A* ≠ Ø, enter the procedure of relaying the external resource of two-hop neighbors in Sec.4.2.2. The procedure continues inviting the two-hop neighbors of cluster head *C_h_* to solve the overloading problem. Otherwise, the dynamic cooperating set planning sub-algorithm is over. *G* is the final cooperating set.

#### 4.2.2. Relaying External Resources of Two-hop Neighbors (ψ^2^_ch_)

In this part we consider adding the 2-hop neighbors of *C_h_*_,_ *ψ^2^_ch_*, to join cluster *G*. Two-hop neighbors are not directly adjacent to *C_h_,* so they cannot serve the users in *C_h_* directly. But they can serve the users in the set of cells A and then increase *β_ch,i_s* where *C^1^_i_* ∈ *A*. By this way, we can increase *β_ch,i_* such that BS *C^1^_i_* is able to offload more *C_h_*’s unserved users. This is what we call “relay external resource’’. Note that the amount of resource that a BS *C^2^_j_* ∈ *ψ^2^_ch_* can transfer is limited to *α_i,j,_* and the traffic demand a BS *C^1^_i_* can offload is limited to *α_ch,i_*. The following describes how to select and add two-hop neighbors of *C_h_* to *G*.

**Step1**. For set *A*, select the C^1^_i__*_ ∈ *A* which has the greatest α_ch,i__*_ in *A*. Consider the adjacent BSs of C^1^_i__*_ and that they need to be the two-hop neighbors of *C_h_*, i.e., *C^2^_j_* ∈ (*ψ^2^_ch_* ∩∩ψ^1^_i__*_), *j* =1, …, | *ψ^2^_ch_* ∩ ψ^1^_i__*_ |. Update *S*= {*C^2^_j_* | *C^2^_j_* ∈ (*ψ^2^_ch_* ∩ψ^1^_i__*_)} and *A* = *A*-*C^1^_i_*. For each *C^2^_j_* ∈ *S*, calculate α_i,j__*_, β_i,j__*_ and *W_j_. W_j_* is defined as follows, which is the weighted average of α_i,j__*_, β_i,j__*_ and F(*C^2^_j_*, *G*),

*W_j_* = *X* ∗ α_i*,j_ + *Y* ∗ β_i,*j_ + (1 − *X* − *Y*) ∗ F(*C^2^_j_*, *G*). (7)

**Step2**. Calculate *π* and select the C^2^_j__*_ in S with the greatest W_j__*_ to *G*, i.e., *G* = *G* + {C^2^_j*_}. Update S = S-{C^2^_j*_}.

**Step3**. If there exist two subsets α^h^_i__*,j*_ ∈ α_i__*,j*_ and α^h^_ch,i__*_ ∈ α_ch,i__*_ such that β_i__*,j*_ ≥ Z^b^_i*,j*_(α^h^_i*,j*_), β_ch,i__*_ + Z_i*_(α^h^_i*,j*_) ≥ Z^b^_ch,i*_(α^h^_ch,i*_), and *π* ≤ Z(α^h^_ch,i*_), which means that C^2^_j__*_ can transfer C^1^_i__*_ sufficient free resource to solve the overloading problem. In this case, set *π* = 0, the whole sub-algorithm terminates, and *G* is the final cooperating set. Otherwise, identify a subset α^h^^’^_i__*,j*_ ∈ α_i__*,j*_, where α^h^^’^_i__*,j*_ = *arg* max{Z_i*_(α^h^_i__*,j*_)| Z^b^_i*,j*_(α^h^_i__*,j*_) ≤ β_i__*,j*_, α^h^_i__*,j*_ ∈ α_i__*,j*_}, then transfer the set of users α^h^^’^_i__*,j*_ to the base station C^2^_j__*_, update α_i__*,j*_, β_i__*,j*_ and β_ch,i__*_, and go to Step 4.

**Step4**. If we can find a subset *α^h^*_ch,i*_ ∈ α_ch,i__*_ such that *π* ≤ Z(α^h^_ch,i*_) and β_ch,i__*_ ≥ Z^b^_ch,i*_(α^h^_ch,i*_) are true, then the whole dynamic cooperating set planning sub-algorithm terminates and *G* is the final cooperating set; if not, identify a subset *α^h’^*_ch,i*_ ∈ α_ch,i__*_, where *α^h^^’^*_ch,i*_ = *arg* max{*Z*(α^h^_ch,i__*_)| *Z^b^_ch,i_*(α^h^_ch,i__*_) ≤ β_ch,i__*_, α^h^_ch,i__*_ ∈ α_ch,i__*_}, then transfer *α^h’^_ch,i_* to the base station C^1^_i__*_ and update α_ch,i__*_, β_ch,i__*_, and *π*. If *S* ≠ Ø, go back to step 2. If *S* = Ø and *A* ≠ Ø go back to step 1. If *S =* Ø and *A* = Ø, then the sub-algorithm is finished and output *G*.

With this dynamic cooperating set planning sub-algorithm, we can effectively offload the overloaded base station by exploiting the free resource of surrounding neighboring base stations. But if the amount of overloaded demand is too much, the cooperating set is still unable to digest the whole excess demand. If this happens, we can further try to adjust the transmission power to increase the spectrum efficiency. In this way, the overloaded base station can service the most users, and the surrounding base stations can help to offload more traffic demand. The details of how to adjust cells’ transmit power to enhance the spectrum efficiency are described in the next subsection.

### 4.3. Transmit Power Allocation (TPA) Sub-Algorithm

Previously, Section 4.2 showed the way of dynamically forming a cooperating set for handling an overload scenario. The cooperating set offloads the traffic request to service more users. The offloading capability corresponds to the size of *α* and the SINRs of users in cell edges. If the SINRs of edge UEs are low, the cooperating BSs can only offload a few UEs. Furthermore, if *α* is small, the cooperating BSs with many available resources hardly offload the traffic request of overloaded cells. In this case, we enhance SINR and *α* by changing the inner and outer transmission power; that is, *TP_i_^inn^* and *TP_i_^out^*, to balance the load. Note that BSs need to satisfy the maximum transmission power constraint (as Equation (5)); moreover, if the system is overloaded, power saving will not be our primary issue. If overloaded, we will mitigate the problem by dynamically composing cooperating set by adjusting *TP_i_^inn^* and *TP_i_^out^*.

**Figure 4:**
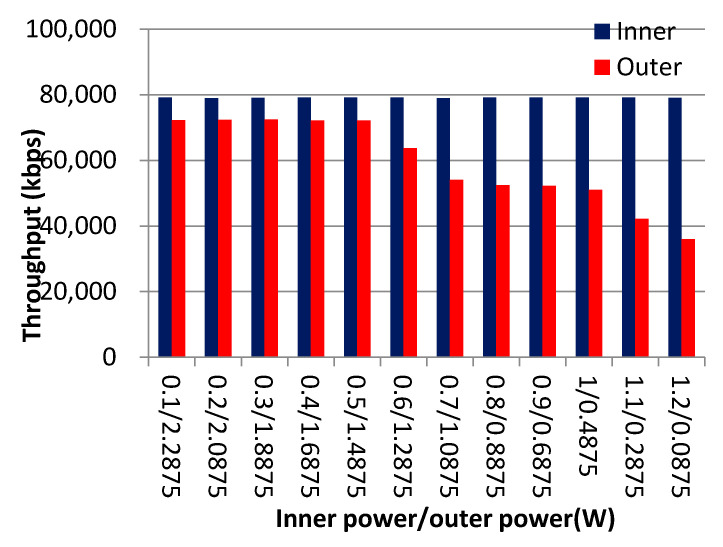
Power allocation VS. inner/outer area throughput.

Figure 4 shows the effect of different *TP_i_^inn^* and *TP_i_^out^* on system throughput. In this figure, *TP_i_^inn^* affects inner region throughput a little, while reducing *TP_i_^out^* harms outer region throughput. The reason is that if the system overloads, the inner region can always select sufficient UEs with better SINR to satisfy their request without considering *TP_i_^inn^*. On the other hand, the *TP_i_^out^* value heavily influences the values of SINR and α of users in cell edges.

Thus, if the network is overloaded after implementing the dynamic cooperating set planning method, we still can change the transmission power. We redefine *C_h_* as *C_1_* and the other base stations in *G* are numbered sequentially from inside to outside cell as *C_i_*, *i* = 2, …, |*G*|. The idea of the transmission power adjustment method is to allocate more transmission power to outer regions so that SINRs and the size of *α* will increase, while the performance of the inner region will not be harmed. Algorithm 1 shows the transmit power allocation sub-algorithm:
**Algorithm 1.** Transmit Power Allocation (TPA) Sub-algorithm1: *S* = *G*2: while *S* ≠ Ø3:  for *TP_i_^inn^* = {*TP_MAX_*/|*S^inn^_i_*|, *TP_MAX_*/|*S^inn^_i_*|-*∆p*, …, 0}4:   find *Ө_i_^inn_new^*
5:   if *Ө_i_^inn_new^ < Ө_i_^inn_old^*6:    *TP_i_^inn^* = *TP_i_^inn^* + *∆p*7:    break8:   else9:    *Ө_i_^inn_old^ = Ө_i_^inn_new^*10:   end if11:  end for12:  TP_i_^out^ = (TP_MAX_-|S^inn^_i_|*TP_i_^inn^)/|S^out^_i_|13:  for *TP_i_^out^* = {*TP_i_^out^*, *TP_i_^out^*-*∆p*, …, 0}14:   find *Ө_i_^out_new^*15:   if *Ө_i_^out_new^ < Ө_i_^out_old^*16:    *TP_i_^out^* = *TP_i_^out^* + *∆p*17:    break18:   else19:    *Ө_i_^out_old^ = Ө_i_^out_new^*20:   end if21:  end for22:  *S* = *S*-*C_i_*23: end while

The algorithm demonstrates how transmission power is allocated to enhance SINR and *α* such that BSs will enhance throughput and radio resource utilization and offload more UEs. In the algorithm, *Ө_i_* is the throughput of *C_i_*, and at the end of the method, the interference level will be changed. Thus, each BS will have to implement fine-tuning. The value of *Δp* will influence the trimming degree.

After the transmission power adjustment, if the overloading condition still exists and some inner regions still have unused radio resources, we can proceed with intra-cell dynamic cell selection, which will be described in Section 4.4. Section 4.4 will further utilize these inner resources and transmit power to dynamically transfer users in outer regions to use the free resource in inner regions, which is called intra-cell dynamic cell selection. As a result, regions will get more free radio resources to help to offload overloaded base stations.

### 4.4. Intra-Cell Dynamic Service Area Selection (ICDSS) Sub-Algorithm

**Figure 5:**
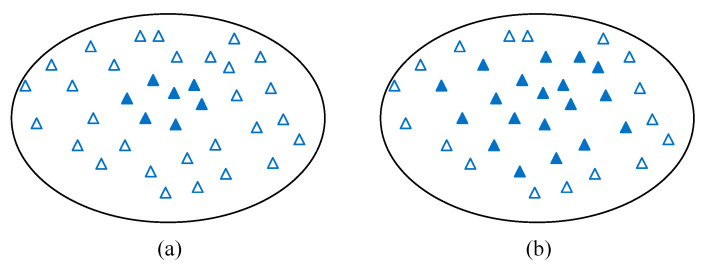
Intra-cell Dynamic Service Area Selection.

Previous subsections discuss how to use the surrounding base stations and radio resources and adapt base station transmission power to reach the most offload. This subsection further explores how to better use every base station’s radio resource (including the inner and outer radio resource) to increase its throughput and enhance the capability of offloading. After executing the procedures proposed in previous subsections, increase *TP_i_^out^* cannot serve more edge users because all the surrounding BSs have run out of their edge resource. To improve the throughput and offloaded capability of the surrounding base stations, this subsection presents the intra-cell dynamic service area selection sub-algorithm. Once the cell’s radio resource is exhausted, but there is still free inner resource and non-zero *α*, we can select some users in the outer region and assign them inner region radio resource. In this way, the utilization of the inner region rises and the free resource of outer regions increases. Therefore, more TTIs can be used to offload. Figure 5 shows an example. As shown in Figure 5a, we can see that the cell center only serves a limited number of users, while most of the users are served by the cell outer area of other cells. Then, in Figure 5b, the cell inner region selects more users such that the cell outer area can get more free resource to help with offloading.

In the previous subsection, only sufficient transmission power is assigned to inner and outer regions. We define the remaining transmission power as *TP_i_^r^* = *TP_MAX_ −* (|*S^inn^_i_*|*TP_i_^inn^ + |*S^out^_i_*|*TP_i_^out^). The following is the proposed intra-cell dynamic service area (selection sub-algorithm).

**Step 1**. If *C_h_* is still overloaded, we initialize *S* = *G-*{*C_h_*}*-ψ^1^_ch_*.

**Step 2**. For each base station *C_j_* ∈ *S*, calculate *α_i,j_* and *TP_j_^r^* (*C_i_* ∈ *G* and *C_i_* ∈ *ψ^1^_ch_*) and execute the following steps.

**Step 2-1**. If *α_i,j_* and *TP_j_^r^* are not 0, assign *TP_j_^r^* to the inner region, i.e., *TP_j_^inn^* = *TP_j_^inn^*+*TP_j_^r^*/|*S^inn^_j_*|. This will enhance the SINR of the inner region, thus more users in the outer region can dynamically select to switch to the inner service area. Therefore, *Ө_j_^inn^* and the free resource of the outer region both increase.

**Step 2-2**. Find a subset of *α^h^_i,j_* ∈ *α_i,j_* such that the transfer of the set of users *α^h^_i,j_* to *C_j_* can get the best spectrum efficiency and help *C_i_* to offload.

**Step 3**. Set *S* = *G* *− C_h_* *− ψ^2^_ch_*. For each base station *C_i_* ∈ *S*, calculate *α_ch,i_* and *TP_i_^r^* and execute the following steps.

**Step 3-1**. If *α_ch,i_* and *TP_i_^r^* are not 0, assign *TP_i_^r^* to the inner region, i.e., *TP_i_^inn^* = *TP_i_^inn^* + *TP_i_^r^*/|*S^inn^_i_*|. This will improve the SINR of the inner region, thus more users in the outer region can dynamically select to switch to the inner service area. As a result, *Ө_i_^inn^* and the free resource of the outer region both also increase.

**Step 3-2**. Find a subset of *α^h^_ch,i_* ∈ *α_ch,i_* such that the transfer of the set of users *α^h^_ch,i_* to *C_i_* can get the best spectrum efficiency and help to offload *C_h_*.

**Step 4**. The base station *C_h_* reallocates its free radio resources, and checks whether it is still overloaded.

After the execution of the dynamic service area selection sub-algorithm, if the system is still overloaded, we can choose to perform the transmission power allocation sub-algorithm (Section 4.3) and the intra-cell dynamic service area selection sub-algorithm again. Through iteratively *TP^inn^*, *TP^out^*, and *TP^r^*, we can reach better spectrum efficiency, serve more users, and realize load balancing. The cost of the proposed scheme in this subsection is that the transferred users (from the outer service area to the inner service area) will consume more energy and radio resource than originally. But, when the system is overloaded, how to utilize the surrounding base stations’ free spectrum and transmission power to help with offloading and load balancing is the first goal. Note that the benefit will decrease as the number of iterations increases. The time required for convergence may be long. An option is to set a threshold to limit the times of iteration. The threshold can be the number of iterations, execution time, or the gain of each iteration.

## 5. Simulation Results

We simulated the performance for our proposed scheme. The system parameters are shown in Table 3. Specifically, there were 19 BSs in the network. The amount of TTIs in a subframe was 50, where 2 TTIs were for control signaling while 48 TTIs were for data transmission. To alleviate the interference problem, we adopted the SFR model. The bandwidth was partitioned into three equal subbands. Two subbands, 32 TTI, were used in the inner area, and one subband, 16 TTIs, was used in the outer area. No adjacent cells used the same subband for the outer area. In addition, we use two ways to generate overloaded UEs: (1) uniform distributed UEs in central BS and (2) exponential distributed UEs in the system. For (1), we first randomly distributed a number of UEs in the system and then generated overloaded UEs uniformly in the central cell only. For (2), we exponentially distributed UEs in the system with a mean 1/μ. These will be cleared in the following subsections.

The proposed scheme compared with the following three schemes: (1) *Single (no CoMP)*: Each base station operated independently and none of them composed a cooperating set. (2) *Static Cooperating Set Planning (SCSP)*: Every three base stations composed a cooperating set, which was done in the initialization stage. After that, all cooperating sets were fixed. (3) *Dynamic Cooperating Set Planning with fixed power configuration (DCSP (0.625))*: DCSP (0.625) used the same dynamic cooperating set planning mechanism as our proposed algorithm, DCSP, but exploited fixed transmit power settings. The transmission power settings are referred to in [26], which suggests the transmission power of the outer area of a cell to be twice that of the inner area. There were four performance metrics: (1) throughput, (2) bandwidth utilization, (3) dropped users, and (4) the size of cooperating set, which are evaluated in the following.

### 5.1. Centralized Overload

In this subsection, 450 users were randomly distributed first in the network, and we generated extra users, *η*, in the cell center to simulate the overloaded scenario. Below, the effect of such a scenario is investigated on different performance metrics.

**Figure 6:**
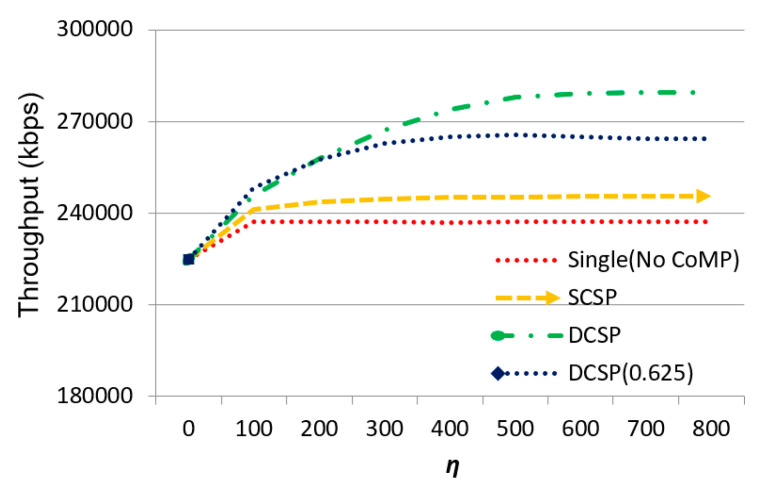
The effect of *η* on throughput.

As shown in Figure 6, the effect of *η* on throughput for all four methods increased, and the throughput increased. Our proposed DCSP was the best and DCSP (0.625) was the second. Comparing DCSP and DCSP (0.625), we could see that through transmitting power adaptation and intra-cell dynamic service area selection, the throughput had a significant improvement. When *η* = 0, the total number of users in the system was 450 users and each base station had 23.7 users in the coverage area on average, so the system was not overloaded yet. All four methods could digest all the traffic demands. When *η* increased, the Single (No CoMP) method was the first to converge (at *η* = 100). This was because each base station operated independently. Once the overloading conditions occurred, it could not digest any excess transmission requirement immediately. SCSP was the second to converge as *η* kept increasing. Although SCSP performed better than Single (No CoMP) because of CoMP, the size of the cooperating set was only 3 and it formed cooperating sets in a static style in which the actual traffic condition was not considered. This is why SCSP was better than Single (No CoMP) but worse than DCSP (0.625) and DCSP. The throughput of DCSP (0.625) and DCSP remained until *η* = 400 and *η* = 600, respectively. This simulation showed that the dynamic cooperating set planning mechanism is very efficient for helping offload the excess data transmission requests hence improve system throughput. Additionally, power distribution increased the overall throughput significantly.

**Figure 7:**
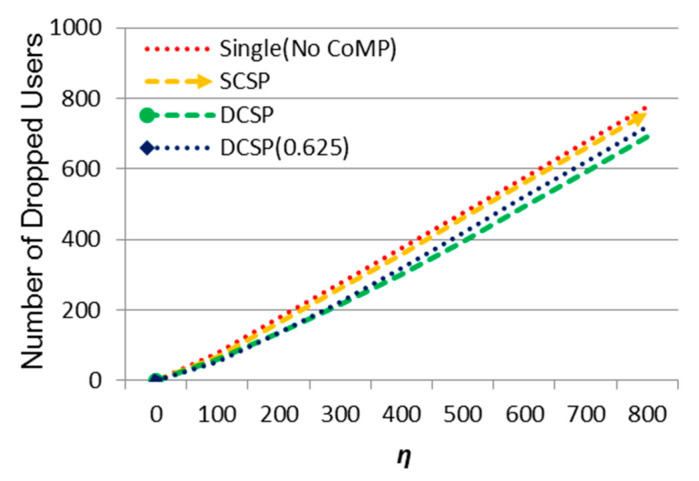
The effect of *η* on the number of dropped users.

Then, the effect of *η* on the number of dropped users is shown in Figure 7. As can be seen, the number of dropped users increased as *η* increases. The proposed methods DCSP and DCSP (0.625) performed the best and the second, respectively. Contrarily, Single (No CoMP) performed the worst due to system overload so that no neighboring cells could support offloading. DCSP dropped more users as *η* increased because system capacity was limited and DCS could help to offload cell edge users but the overloaded users in the cell center area could only be serviced by the central cell itself.

**Figure 8:**
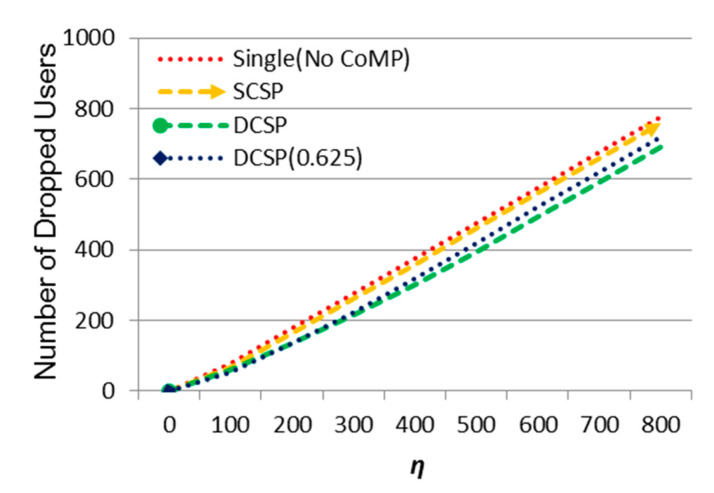
The effect of *η* on average cluster size.

Now, we observed the effect of *η* on average cluster size in Figure 8. It showed that as *η* increased, the average cluster size increased. DCSP and DCSP (0.625) dynamically increased the size of the cooperating set based on the real traffic scenario for helping offload. Since DCSP adapts power to efficiently use power and bandwidth compared to DCSP (0.625), the cluster size of DCSP was smaller than DCSP (0.625). So, DCSP does not have to invite too many 2-hop neighbors to join the cooperating set. Additionally, we found that if there was a large quantity of users located in the overlap region (i.e., *α_i,j_* and *α_ch,i_* is large), our proposed method could benefit from this and improve the system throughput a lot.

**Figure 9:**
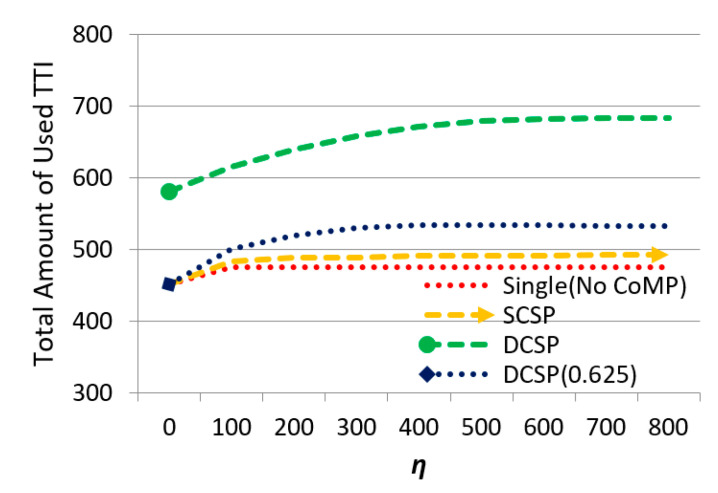
The effect of *η* on the total amount of used TTI.

Consequently, the effect of *η* on the total amount of used TTI (bandwidth utilization) is shown in Figure 9. When *η* increased, the total amount of used TTI increased. Our DCSP scheme performed the best. Figure 9 shows that bandwidth utilization of DCSP was much better than the other three schemes. This presents that the proposed intra-cell dynamic service area selection (Dynamic Serving-area Selection) and transmission power adjustment (Transmit Power Allocation Sub-algorithm) could effectively utilize the bandwidth and power resource, which also enhanced throughput (as shown in Figure 6).

**Figure 10:**
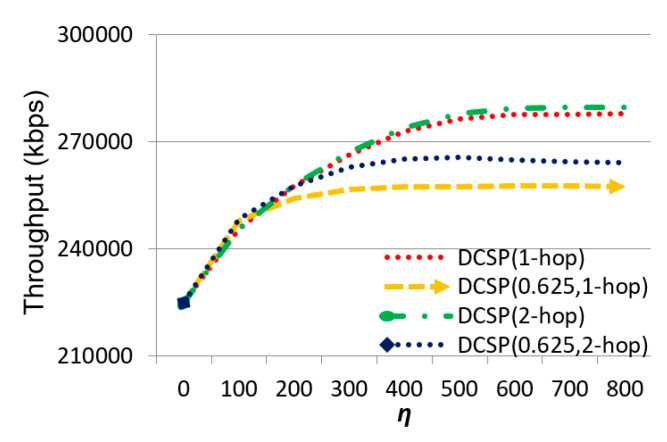
The effect of *η* on throughput (1-hop neighbors as candidates VS. 1-hope and 2-hop neighbors as candidates).

In the proposed dynamic cooperating set planning sub-algorithm, it could include up to 2-hop neighbors to join the cooperating set. In this experiment, we evaluated the performance difference when different hop counts of neighbor cells were considered. We compared four schemes: DCSP(1-hop), DCSP, DCSP (0.625,1-hop), and DCSP (0.625,2-hop), where DCSP(1-hop) and DCSP (0.625,1-hop) could ask up to 1-hop neighbors (2-hop neighbors were not allowed) to join the cooperating set when forming a dynamic cooperating set. As shown in Figure 10, we could see that when more neighbor cells can be considered in forming the dynamic cooperating set, the throughput performance was better. This was because more cells can help with offloading more overloaded traffic demands. Experiment results showed that DCSP > DCSP(1-hop) > DCSP (0.625,2-hop) > DCSP (0.625,1-hop).

### 5.2. Exponential Random Distribution Overload

**Figure 11:**
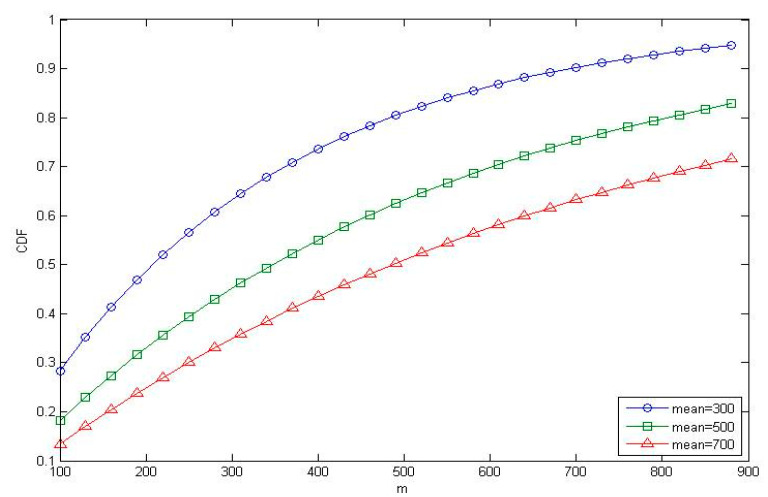
The effect of m on CDF.

In this subsection, we applied the exponential distribution model to generate user (ᶆ). Figure 11 shows that when the mean value of the exponential distribution was high, so was the user distribution in a uniform manner (mean = 700), while the lower mean value meant that the users were in a more concentrated distribution (mean = 300). In the following, we applied the user mean = 300 to generate an overloading situation like a concert or baseball game, where users will gather in a concentrated area.

**Figure 12:**
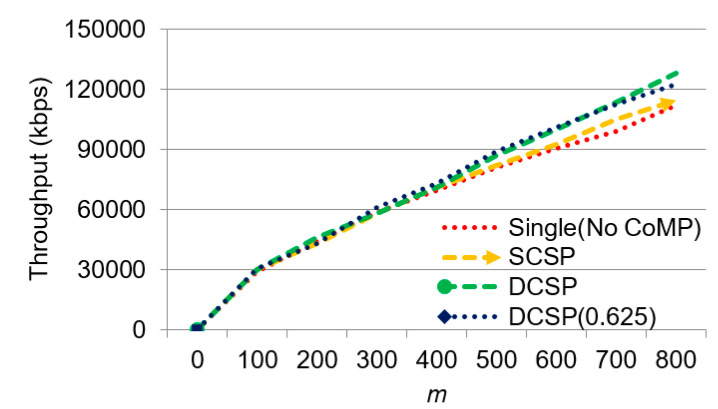
The effect of ᶆ on throughput.

We can now observe the effect of m on throughput in Figure 12. We can see that when m increased, the throughput increased. Single had the lowest throughput because each base station operated independently. When the overloading occurred, it could not digest the excess transmission requirements. Although SCSP had the composition of collaboration collection, the composition had no flexibility; therefore, the throughput could not be increased too much. DCSP (0.625) and DCSP were the best two methods, and both of which did not begin to have difference until after ᶆ = 700, which meant that a collection in a dynamic collaborative manner could indeed be effective in helping offload its base station’s overload excess demand for data transmission, thus improving system throughput.

**Figure 13:**
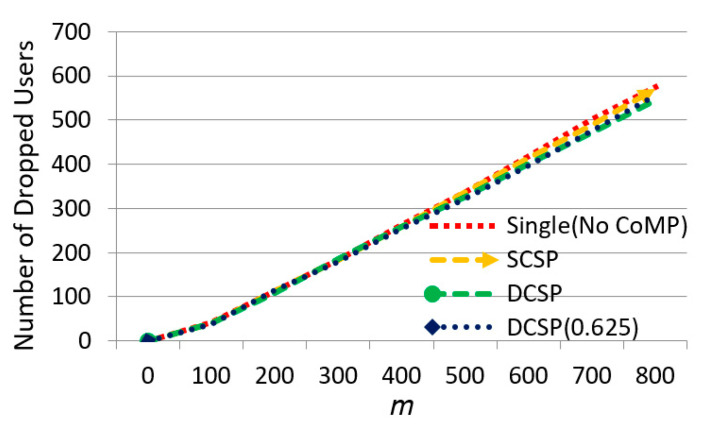
The effect of ᶆ on the number of dropped users.

Then, we observed the effect of m on the number of dropped UEs in Figure 13. We could see that when m increased, the number of dropped UEs increased. Our proposed methods DCSP and DCSP (0.625) had the best performance. When the system began to overload, some users were in the center area and other users were in the edge area. Although cell collaboration within the collection could help to offload overloaded users, if the overloaded users were not in the edge area, when the resource in the center area ran out, these users would be dropped.

**Figure 14:**
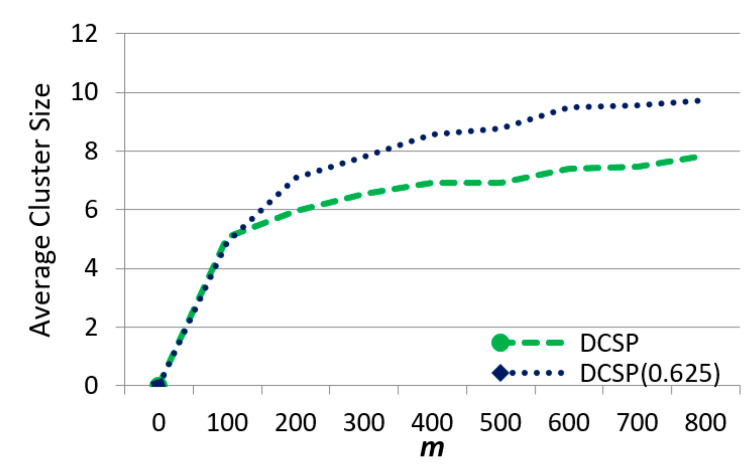
The effect of ᶆ on average cluster size.

Now, we observed the effect of m on average cluster size in Figure 14. The DCSP collaboration had a smaller set size because it also applied the power allocation scheme for better bandwidth usage, and the idle resources could be utilized. DCSP (0.625) could not effectively use resources in the center, and with the increase ᶆ, the neighboring base stations ran out of resources in the edge areas and thus could not help with offloading.

**Figure 15:**
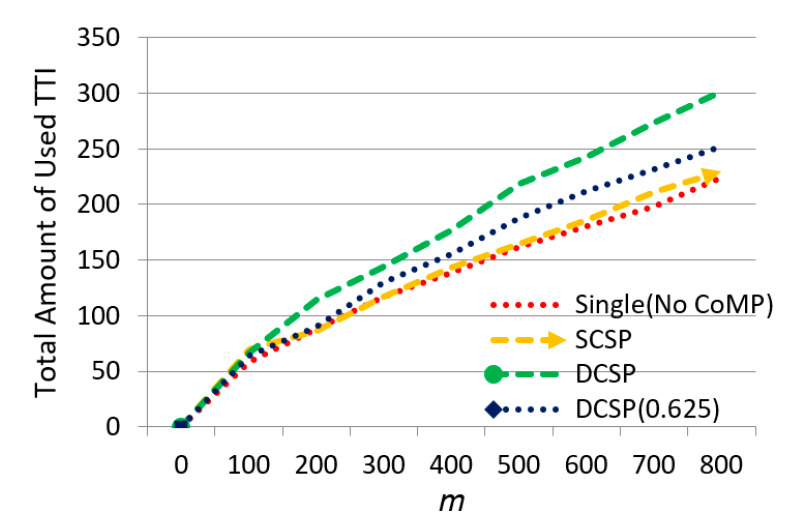
The effect of ᶆ on the total amount of used TTI.

Then, we observed the effect of m on the total amount of used TTI in Figure 15. We could see that when m increased, the total amount of used TTI increased. DCSP had the most efficient resource usage when the base station was idle because our proposed Intra-cell dynamic service area selection algorithm effectively adjusted the power allocation and spectrum usage, and when the system was overloaded, the idle resources in the edge areas could be effectively used, which helped with offloading base station overload and improved the overall throughput and the utilization of TTI.

**Figure 16:**
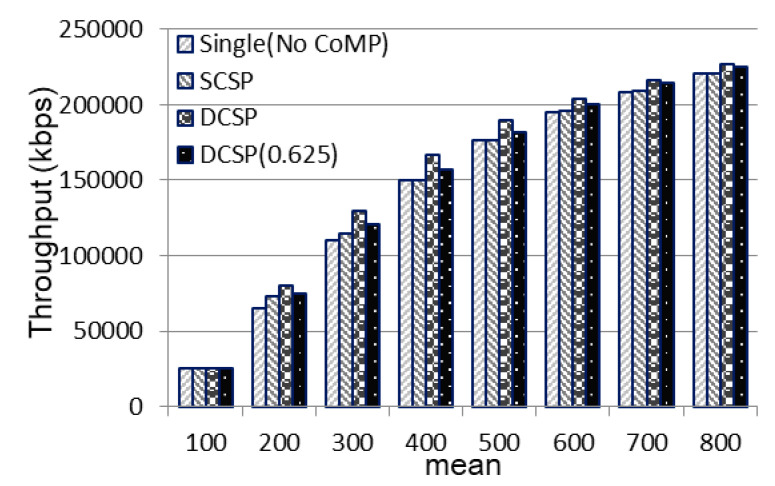
The effect of mean on throughput.

Finally, we observed the effect of the mean on the throughput of all four methods in Figure 16. When mean = 100, the throughput of all four methods was the same because the number of users was too concentrated in the center area of the base station, and the base station could not apply the offload feature. When mean = 200–600, our proposed method was better than the other methods. When the mean ≥700, because the users were evenly distributed (Figure 5a), when the center area started to overload and the surrounding base stations were saturated, the throughput of the system of the four methods was similar but compared to other methods, our methods DCSP and DCSP (0.625) were still the best.

## 6. Conclusions

We propose a distributed energy-efficient, dynamic cooperating set planning method in B4G/5G networks for DL CoMP in this paper. By exploiting a dynamic cooperating set, dynamic inter-/intra-cell selection, and power adaptation, the scheme reaches a better radio resource and energy utilization such that both the system throughput and load-balancing are all significantly improved. During off-peak hours, the proposed scheme reduces unnecessary power consumption with improvements in energy-saving and radio resource utilization. During peak hours and/or when a large-scale event occurs with crowds gathering, the proposed scheme can offload excessive traffic demands by dynamically composing the cooperating set based on real traffic requests, adapting cells’ transmission power, and selection of the dynamic service areas. This efficiently enhances radio resource utilization and energy efficiency, thus significantly enhancing system performance. Simulation results show that our scheme has better system throughput, fewer dropped users, and higher radio resource utilization, and efficiently helps with offloading and enhances system energy efficiency. For future work, we will further consider beamforming techniques and apply machine learning solutions to solve the dynamic set planning issue so as to potentially enhance the performance in terms of resource utilization and efficiency.

## Figures and Tables

**Table 1:** MCS and required received Signal-to-Interference-plus-Noise Ratio (SINR).

Modulation	Code Rate	SINR(dB)
16QAM	1/2	7.9
	2/3	11.3
	3/4	12.2
	4/5	12.8
64QAM	2/3	15.3
	3/4	17.5
	4/5	18.6

**Table 2:** Notations used in the paper.

Notation	Definition
*F_i_*	The *i-th* frequency subband
*F_Ci_^outer^*	The subbands used in the outer area of Cell_i_
*F_Ci_^inner^*	The subbands used in the inner area of Cell_i_
*M*	The number of cells
*N*	The number of UEs
*δ_i_*	Average data rate for UE_i_ (bits/s)
*r_j_*	UE_j_’s data request
*TP_i_^inn^*	The inner transmission power of BS i (watt/TTI)
*TP_i_^out^*	The outer transmission power of BS i (watt/TTI)
*TTI^inn^_(i,j)_*	The needed resource for user j in the inner region
*TTI^out^_(i,j)_*	The needed resource for user j in the outer region
*Eff(x)*	The number of data bits that a TTI can carry with MCS x
*TTI_i_^inn^*	The total requirements of BS *j* for inner regions
*TTI_i_^out^*	The total requirements of BS *j* for outer regions
*Thr^inn^_lw_*	The lower threshold of *TTI_i_^inn^*
*Thr^inn^_up_*	The upper threshold of *TTI_i_^inn^*
*Thr^out^_lw_*	The lower threshold of *TTI_i_^out^*
*Thr^out^_up_*	The upper threshold of *TTI_i_^out^*
*S_i_^inn^*	The set of used TTIs in inner regions
*S_i_^out^*	The sets of used TTIs in outer regions
*ψ^1^_Ch_*	The adjacent BSs to the overloaded BS (*C_h_*)
*N^1^_i_*	The *i-th* direct neighboring cell of the overloaded BS (*C_h_*)
*α_m,n_*	The set of users which is served by BS *m* and is also covered by BS *n*
*β_u,v_*	The amount of free resource in the cell edge of BS *v* which can be provided to BS *u*
*F(C^1^_i_, G)*	The number of adjacent edges between BS *C^1^_i_* and *G*
*π*	The total amount of overloaded traffic demand of *C_h_*
Z*(α_ch,i_)*	The total required amount of radio resource in C*_h_* for the set of users *α_ch,i_*
*ψ^2^_ch_*	The 2-hop neighbors of *C_h_*
*TP_MAX_*	The maximal transmission power
*TP_i_^r^*	The remaining transmission power

**Table 3:** System parameters.

Parameter	Value
system bandwidth	10 MHz
distance between two neighboring	866 m
transmission power of a BS and a UE	46 dBm, 23 dBm
antenna height of a BS	32 m
inner area of each cell	2/3
average data rate of each UE	500 kbps

## Data Availability

The data used to support the findings of this study are included within the article.
